# Risk of Parkinson's Disease in the Users of Antihypertensive Agents: An Evidence from the Meta-Analysis of Observational Studies

**DOI:** 10.1155/2016/5780809

**Published:** 2016-07-19

**Authors:** Amarnath Mullapudi, Kapil Gudala, Chandra Sekhar Boya, Dipika Bansal

**Affiliations:** Department of Pharmacy Practice, National Institute of Pharmaceutical Education and Research, SAS Nagar, Punjab 160062, India

## Abstract

*Background*. Antihypertensive agents have been shown to inhibit oxidative stress and inflammatory response and thus neuroprotection in Parkinson's disease (PD). Epidemiological evidence suggests inconsistency between use of antihypertensives and risk of PD. This study is aimed to examine the association between antihypertensive use and risk of PD.* Methods*. Literature search in PubMed, EMBASE, and PsycINFO database was undertaken through February 2012 looking for observational studies evaluating the association between antihypertensive drug use and risk of PD. Before meta-analysis, the studies were evaluated for publication bias and heterogeneity. Pooled relative risk (RR) estimates and 95% confidence intervals (CIs) were calculated using random-effects model (DerSimonian and Laird method). Subgroup analyses and sensitivity analysis were also performed.* Results*. Seven relevant studies including a total of 28,32,991 subjects were included. Pooled RR of overall use of antihypertensive agents was found to be 0.95 (95% CI 0.84–1.05). A significant reduction in the risk of PD was observed among users of calcium channel blockers (RR 0.82, 95% CI 0.71–0.93). Significant heterogeneity (*I* ^2^ = 76.2%) but no publication bias was observed.* Conclusions*. Overall use of antihypertensive agents showed no significant association with the risk of PD. CCBs provided significant protective role. However, studies with large sample size and dose relationships are required to strengthen our hypothesis.

## 1. Introduction

Parkinson's disease (PD) is the most common neurodegenerative disorder, which is more prevalent in elderly population [1]. The pathogenesis of PD involves degeneration of dopaminergic neurons in the substantia nigra of the midbrain region, which results in the imbalance of dopamine levels leading to the development of PD symptoms which include resting tremors, rigidity, bradykinesia, and postural instability [2]. Animal models have reported that oxidative stress, excitotoxicity, and alpha-synuclein aggregation are the major factors responsible for the degeneration of dopaminergic neurons [3].

Antihypertensive agents majorly include calcium channel blockers (CCBs), beta blockers, angiotensin converting enzyme inhibitors (ACE inhibitors), and angiotensin II receptor blockers (ARBs). These drugs are used to treat hypertension and cardiovascular diseases [4]. Evidence from preclinical studies showed that CCBs, beta blockers, ACE inhibitors, and ARBs may have neuroprotective effect by inhibiting calcium overload, nitric oxide, reactive oxygen species (ROS), tumor necrosis factor-*α*, and interleukin-1 *β* synthesis which are responsible for neurotoxicity and degeneration of dopaminergic neurons [3].

Observational studies have shown an association between antihypertensive agents use and PD risk. But the results are conflicting [5–11]. In the present meta-analysis, we aimed to assess the association between the use of antihypertensives and the risk of PD.

## 2. Materials and Methods

### 2.1. Literature Search

A comprehensive literature search was performed independently by two authors (KG and MA) in databases including PubMed, PsycInfo, and Cochrane library till August 2015 by using the English language and humans as filters to find out the relevant studies. Search terms include “Antihypertensive drugs”, “antihypertensive agents”, “calcium antagonists”, “Angiotensin receptor blockers”, “Angiotensin converting enzyme inhibitors”, “Beta blockers”, and “Parkinson's disease”. We have also searched the bibliographies of the relevant articles. We have screened titles and abstracts of the search results for eligibility and read the full text if required and included studies in the present meta-analysis as per eligibility criteria.

### 2.2. Inclusion and Exclusion Criteria

We have screened titles and abstracts of the search results for eligibility and read the full text if required and included studies in the present meta-analysis as per eligibility criteria. Studies were included if they met the following criteria: (1) clearly defined PD; (2) defined exposure as the use of antihypertensive agents; (3) either observational (case-control and cohort) or experimental studies reporting an association between use of antihypertensive agents and risk of PD; (4) studies reporting effect estimates with confidence intervals (CIs). We have excluded the articles, if they were reviews, letters to the editor without original data, editorials, case reports, or clinical trials. Studies that were not published in English language were also excluded.

### 2.3. Data Extraction

After retrieving the relevant articles from the databases, two authors (MA, KG) independently extracted the data from included studies. The following information was extracted from each study: (i) first author name, year of publication, and country; (ii) study design; (iii) number of subjects and PD cases and size of the cohort; (iv) effect estimates and 95% confidence intervals; (v) assessment of antihypertensive exposure and assessment of PD; (vi) control of confounding factors, if any; and other relevant information.

### 2.4. Quality Assessment

Two authors (MA, BC) assessed the quality of all included studies. Newcastle-Ottawa Scale (NOS) was utilized to assess the quality of the included observational studies. Scores were allotted to every study with the consideration of selection, comparability, and outcome/exposure [12]. Studies with a score of 9 points were considered as high-quality studies, whereas 6–8 points reflect the medium quality and below 6 points reflect low-quality studies.

### 2.5. Statistical Analysis

We pooled the risk ratios (RR) of all included studies to get an overall effect estimate with 95% confidence interval (CI). We have used statistical software, that is, Stata, for pooling the data [13]. The pooled studies were analysed for the heterogeneity using Cochrane *Q* and *I* ^2^ statistics. For the Cochrane *Q* statistics a *P* value > 0.10 and for *I* ^2^ a value of > 50% were considered statistically significant for heterogeneity [14]. If any significant heterogeneity was present among the included studies we have chosen random-effects model over fixed effects model. Fixed effects model was applied otherwise.

Publication bias was assessed initially by visual inspection of the funnel plot and further confirmed by Egger's test. A *P* value of more than 0.1 for Egger's test indicates the presence of publication bias [15]. Duval and Tweedie nonparametric trim and fill method was applied if significant publication bias exists [16].

Subgroup analysis was performed to assess the factors responsible for heterogeneity among the studies in reporting risk ratios. Subgroup analysis was performed based on the class of drugs, study design, gender, age group, and quality of the study.

We have also performed sensitivity analysis to assess the impact of single studies on pooled effect estimate to ensure robustness of results.

## 3. Results

### 3.1. Search Results

**Figure 1:**
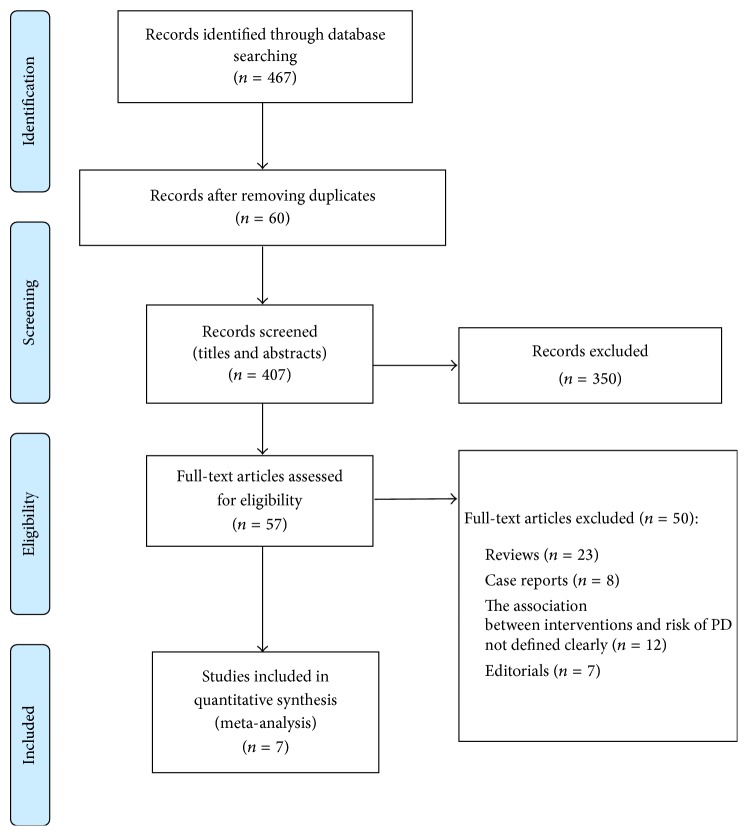
PRISMA flow chart representing the process of selection of studies.

Systematic literature search (Figure 1) in the databases (PubMed, PsycInfo, and Cochrane library) yielded 467 articles. After screening the titles and abstracts of the studies, we have excluded 60 articles found as duplicates. Further screening of 407 articles has shown that search results included animal models, uncontrolled and nonrandomized trials, reviews, and case series and reports which were excluded (*n* = 350). 57 studies were read as full papers. After detailed evaluation of the remaining 57 articles, 44 studies were ineligible as there were reviews (*n* = 23), case reports (*n* = 8), and editorials (*n* = 7). Among the excluded studies of 44, 12 studies did not clearly mention the association between antihypertensives and risk of PD. Finally, 07 studies which met the eligibility criteria were included in the analysis.

### 3.2. Study Characteristics

The study characteristics included in the meta-analysis (*n* = 7) are shown in Tables 1 and 2. All the included studies were observational and published within a period of seven years (2007 to 2014). Among them, 4 were cohort [5–8] and 3 were case-control in design [9–11]. Sample size of the studies ranged from 556 to 25,73,281 and the follow-up period of studies ranged between 4 and 16 years.

### 3.3. Quality Assessment

Quality of included cohort studies is assessed by examining selection, comparability, and outcome. We found that Lee et al. [5] and Pasternak et al. [6] were methodologically high-quality studies whereas Simon et al. [7] and Louis et al. [8] studies were of medium quality as shown in Table 3.

Similarly, we have also assessed the quality of case-control studies (Table 4) by examining selection, comparability, and exposure. We found that US-based study by Ton et al. [11] is a high-quality study whereas studies by Becker et al. [9] and Ritz et al. [10] were medium quality studies according to the rating given by NOS.

### 3.4. Overall Antihypertensive Agents and PD Risk

**Figure 2:**
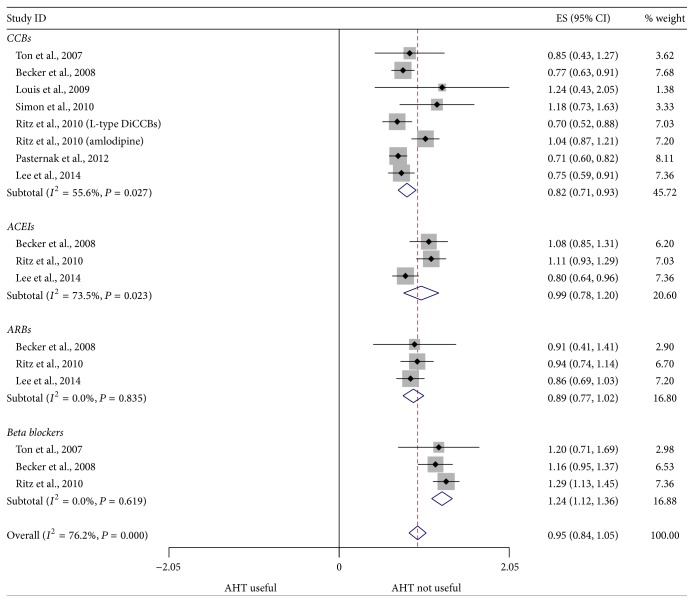
Forest plot showing a combined effect estimates of the risk ratios and 95% confidence intervals. The horizontal line indicates 95% CI and the diamond indicates overall pooled estimate. AHT: antihypertensive; ES: effect size; CI: confidence intervals.

Since significant heterogeneity (*P* = 0.00; *I* ^2^ = 76.0%) was observed in the included studies, random-effects model was chosen over a fixed effects model. Our study results showed that pooled effect estimate was found to be RR 0.95, % CI 0.84–1.05. This confirms that the overall use of antihypertensive agents provided no protection against the risk of PD. The RR of individual study and all studies together is shown in Figure 2.

### 3.5. Calcium Channel Blockers and Risk of PD

The pooled estimate of 7 studies on the association between use of CCBs and PD shows a significant reduction in the risk of PD in the users of CCBs (RR 0.82, 95% CI 0.71–0.93). A significant heterogeneity was found among the studies of CCBs (*P* = 0.02; *I* ^2^ = 55.6%); hence, we have chosen random-effects model.

### 3.6. Subgroup Analysis

Subgroup analysis was performed by study design, class of drugs, gender, and quality of study as presented in Table 5. We found a significant difference between studies according to study design; when compared to case-control studies (RR 0.83, 95% CI 0.67–1.00) cohort studies (RR 0.74, 95% CI 0.65–0.83) showed a higher risk reduction of PD in CCB users. Both the classes of CCBs, that is, dihydropyridine calcium channel blockers (DiCCB) (RR 0.78, 95% CI 0.66–0.98) and non-DiCCB (RR 0.70, 95% CI 0.58–0.83), were found to be reducing the risk of PD. We also found a significant reduced risk of PD in women (RR 0.67, 95% CI 0.69–0.89), contrary to men (RR 0.79. 95% CI 0.57–0.76). Sensitivity analyses performed by excluding one study at a time showed that the main results were robust (RR 0.82, 95% CI 0.71–0.93).

### 3.7. ACE Inhibitors and Risk of PD

The pooled effect estimate of 3 studies that reported on the association between use of ACE inhibitors and risk of PD showed no significant association (RR 0.99, 95% CI 0.78–1.20). A significant heterogeneity was found among the studies of ACE inhibitors (*P* = 0.02; *I* ^2^ = 73.5%); hence we have chosen random-effects model.

Subgroup analysis (Table 6) for ACE inhibitor use showed no significant gender difference in the association, that is, for females (RR 0.76, 95% CI 0.56–1.05) and males (RR 0.88, 95% CI 0.63–1.23). Sensitivity analyses had shown that the main results were robust (RR 0.99, 95% CI 0.78–1.20).

### 3.8. ARBs and Risk of PD

Pooling of effect estimates of 3 studies showed an insignificant reduction (RR 0.89, 95% CI 0.77–1.02) in risk of PD among ARB users. As no significant heterogeneity was found among the studies of ARBs (*P* = 0.36; *I* ^2^ = 0.00), fixed effects model was chosen. Sensitivity analyses had shown that the main results were robust (RR 0.89, 95% CI 0.77–1.02).

### 3.9. Beta Blockers and Risk of PD

Pooling of effect estimates of 3 studies showed a significant increase in the risk of PD in the users of beta blockers (RR 1.24, 95% CI 1.12–1.36). As no significant heterogeneity was found among the studies (*P* = 0.69; *I* ^2^ = 0.00), fixed effects model was chosen. We could not perform subgroup analysis for the studies of beta blockers due to lack of data for individual subgroups. Sensitivity analyses had shown that the main results were robust (RR 1.24, 95% CI 1.12–1.36).

### 3.10. Assessment of Publication Bias

Inspection of Begg's funnel plot did not suggest the presence of publication bias. Further, it was also confirmed by Egger's test (*P* = 0.21).

## 4. Discussion

Our meta-analysis of 7 studies did not find an overall significance on the association of PD risk with use of antihypertensive agents. Although constant use of antihypertensives as a class like CCBs, ACE inhibitors, ARBs, and beta blockers showed an association with risk of PD, we made few interesting observations in our analysis. Firstly, the pooled risk ratio in the users of CCBs from 7 studies showed that there is reduced risk of PD by 18% (RR 0.82, 95% CI 0.71–0.93). An increased risk reduction in women (RR 0.67, 95% CI 0.69–0.89) CCB users was observed as compared to men (RR 0.79, 95% CI 0.69–0.89). Similarly, dihydropyridine calcium channel blockers (DiCCBs) (RR 0.70, 95% CI 0.66–0.89) have shown a significantly higher risk reduction as compared to Non-DiCCBs (RR 0.78, 95% CI 0.58–0.83). Secondly, for the pooled risk ratio in the users of ACE inhibitors (RR 0.99, 95% CI 0.78–1.20) and ARBs (RR 0.89, 95% CI 0.77–1.02), we cannot conclude the hypothesis that ACE inhibitors and ARBs reduce the risk of PD.

Thirdly, the pooled risk ratio in the users of beta blockers (RR 1.24, 95% CI 1.12–1.36) from 3 studies has shown a significant increase 24% in the risk of PD as compared to nonusers. However, it is difficult to conclude the hypothesis regarding beta blockers due the fact that the included studies were case-control studies having less sample size.

Epidemiological evidence has shown heterogeneity in the association of use of CCBs and risk of PD. A recently published retrospective cohort study [5] in Taiwan found 29% reduction in risk of PD (RR 0.71, 95% CI 0.57–0.90) among DiCCB users but not in non DiCCB users (RR 0.73, 95% CI 0.52–1.04). These results are consistent with the result of a large nationwide cohort study [6] (RR 0.71, 95% CI 0.60–0.82). Another two cohort studies [7, 8] investigating the association concluded no observed association, (RR 1.24, 95% CI 0.43–2.05) and (RR 1.18, 95% CI 0.73–1.63).

Similarly, case-control studies conducted to assess the association have also shown heterogeneous results. A population based case-control study [11] of 206 cases did not observe any clear association between PD risk and CCBs (RR = 0.85; 95% CI 0.43–1.27), either for constant use or in terms of length, dose, number of dispensed prescriptions, or pattern of use. Another two case-control studies, Becker et al. [9] (RR = 0.77 (95% CI 0.63–0.91)) and Ritz et al. [10] (RR = 0.70 (95% CI 0.52–0.88)), have reported significant protective role of CCBs and reduced risk of PD.

Parkinson's disease is a neurodegenerative disorder which involves complex pathophysiological mechanisms. The exact mechanisms involved are unknown. Several animal studies have reported that pathophysiology of PD involves complex mechanisms like *α*-synuclein aggregation and oxidative stress leading to neuronal degeneration via calcium overload and apoptosis [17]. Blockade of calcium channels in the central nervous system (CNS) might show protective effect on neurons which may decrease neurodegeneration [18]. It was reported that antagonists of voltage dependent calcium channels are effective in protecting CNS neurons against excitotoxicity mediated cell death and reducing the aggregation of alpha-synuclein particles [19]. Animal studies suggested that DiCCBs have shown a neuroprotective effect in the areas of nigra and striatal regions of brain [20]. We found a reduced risk of PD among women in subgroup analysis. Gender specific results were given in three studies [6, 7, 9]. On pooling these, it was found that the effect estimate of protective role of CCBs was significantly higher in women (RR = 0.67, 95% CI 0.57–0.76). Our results are consistent with the individual study results of Becker et al. (RR 0.66, 95% CI 0.47–0.93) and Pasternak et al. (RR = 0.67, 95% = 0.53–0.85). It was suggested that women have delayed development of PD due to the presence of higher physiological dopamine levels, possibly due to the activity of oestrogens [21]. This could be a possible reason for low risk of PD in women. However, further studies are required for better understanding of the role of CCBs and their protective effects specifically to gender.

Animal studies have reported that ACE inhibitors have neuroprotective effect which might be helpful in reducing the risk of PD. The renin-angiotensin system (RAS) plays an important role in the initiation and continuation of inflammation and oxidative stress in the dopaminergic neurons [22]. In CNS, angiotensin converting enzyme (ACE) is responsible for the conversion of angiotensin-1 (AT_1_) to angiotensin-2 (AT_2_). Activation of AT_2_ receptor stimulates Nicotinamide adenine dinucleotide phosphate oxidase which eventually increases the production of ROS; these ROS may cause degeneration of dopaminergic neurons by oxidation of proteins and lipids of the cell membrane [23]. Studies had reported that animals treated with ACE inhibitors showed a significant decrease in the reduction of dopaminergic neurons in substantia nigra. Sulfhydryl group containing ACE inhibitors are believed to show neuroprotection by scavenging free radicals [24]. It was also observed that blockade of the AT_1_ receptor by ARBs led to a significant decrease in neurodegeneration [25]. Studies with large sample sizes and drug-dose relationships are required to support the hypothesis.

Three case-control studies had reported that use of beta blockers may increase the odds of PD risk [9–11]. In humans, it was documented that there is loss of norepinephrine neurons in the locus coeruleus in patients with PD [26]. Beta blockers compete for the available receptor sites which may result in the reduction of neurotransmission of norepinephrine in the brain which causes the aggregation of alpha-synuclein resulting in the loss of dopaminergic neurons [27]. In our analysis, findings support the above hypothesis which has shown that there is an increased risk of PD among the users of beta blockers.

Our meta-analysis has some limitations to be addressed. Firstly, the studies included in the analysis were all observational studies which may lead to recall bias. Secondly, there is a limited availability of studies and data on dose and duration relationship which restrains us from performing a subgroup analysis on dose and duration of individual antihypertensive agents. Thirdly, the follow-up periods of most of the studies are not more than a decade which may require priorly the diagnosis of PD which could provide strength to the analysis.

## 5. Conclusions

We found that overall antihypertensive agents use does not appear to modify the risk of PD, but subgroup analysis revealed that use of CCBs as a subclass may have a protective effect in lowering the risk of PD. It was also found that there is an increased risk of PD in the users of beta blockers. Further studies on dose and duration relationships and studies with long term follow-up are needed to confirm the use of antihypertensives in the management of PD.

## Figures and Tables

**Table 1:** Characteristics of the cohort studies.

Author, year (country)	Cohort name	Cohort size	Follow-up period	Number of antihypertensive users	Number of PD cases
Lee et al., 2014 [5] (Taiwan)	NR	65001	4.6 years	650001	650001
Pasternak et al., 2012 [6] (Denmark)	NR	25,73,281	8 years	202836	57111
Simon et al., 2010 [7] (USA)	Nurses' health study and health professionals follow-up study	1,71,335	16 years	3826	421
Louis et al., 2009 [8] (Spain)	Neurological disorder in central Spain	3942	4 years	NR	NR

NR: not reported.

**Table 2:** Characteristics of case-control studies.

Author, year (country)	Period of recruitment	Study size	Number of PD patients	Assessment of antihypertensive use	Assessment of PD
Ritz et al., 2010 [10] (Denmark)	2001–2006	11582	1931	National Pharmacy Database	Hospital records
Becker et al., 2008 [9] (UK)	1994–2005	7274	3637	General practice research database	General practice research database
Ton et al., 2007 [11] (USA)	1992–2002	556	191	Medical records	Medical records and cardinal signs

**Table 3:** Newcastle-Ottawa Scale to assess the quality of cohort studies.

Study	Selection	Comparability	Outcome	Total score	Quality of the study
Lee et al., 2014 [5]	*∗∗∗∗*	*∗∗*	*∗∗∗*	9	High quality
Pasternak et al., 2012 [6]	*∗∗∗∗*	*∗∗*	*∗∗∗*	9	High quality
Simon et al., 2010 [7]	*∗∗∗*	*∗∗*	*∗∗*	7	Medium quality
Louis et al., 2009 [8]	*∗∗∗*	*∗∗*	*∗∗∗*	8	Medium quality

A study can be awarded a maximum of 4 stars for selection, a maximum of 2 stars for comparability, and a maximum of 3 stars for outcome.

**Table 4:** Newcastle-Ottawa Scale to assess the quality of case-control studies.

Study	Selection	Comparability	Outcome	Total score	Quality of the study
Becker et al., 2008 [9]	*∗∗∗∗*	*∗∗*	*∗∗∗*	9	High quality
Ritz et al., 2010 [10]	*∗∗∗∗*	*∗∗*	*∗∗∗*	9	High quality
Ton et al., 2007 [11]	*∗∗∗*	*∗∗*	*∗∗*	7	Medium quality

A study can be awarded a maximum of 4 stars for selection, a maximum of 2 stars for comparability, and a maximum of 3 stars for exposure.

**Table 5:** Overall effect estimates for Parkinson's disease and calcium channel blockers use according to subgroups.

Characteristics	Number of studies	RR (95% CI)	*P* value	Cochrane *Q* value	*I* ^2^ value
All studies	7	0.82 (0.71–0.93)	0.016	0.027	55.678

Study design					
Cohort	4	0.74 (0.65–0.83)	<0.001	0.144	44.605
Case-control	3	0.83 (0.67–1.00)	0.111	0.035	65.022

Class of CCBs					
DiCCB	4	0.70 (0.66–0.89)	0.032	0.016	67.027
Non DiCCB	3	0.78 (0.58–0.83)	0.013	0.745	0.000

Gender					
Men	4	0.79 (0.69–0.89)	0.080	0.175	39.499
Women	3	0.67 (0.57–0.76)	0.071	0.954	0.000

Quality					
High	3	0.71 (0.63–0.79)	0.001	0.793	0
Medium	4	0.89 (0.71–1.07)	0.272	0.026	65.744

**Table 6:** Overall effect estimates for Parkinson's disease and Angiotensin converting enzyme inhibitors use according to subgroups.

Characteristics	Number of studies	RR (95% CI)	*P* value	Cochrane *Q* value	*I* ^2^ value
All studies	3	0.98 (0.77–1.2)	0.01	0.023	73.422

Study design					
Cohort	1	0.80 (0.64–0.96)	0.12	0.840	0.000
Case-control	2	1.09 (0.64–1.24)	0.14	1.000	0.000

Gender					
Men	1	0.88 (0.63–1.23)	NA	NA	NA
Women	2	0.76 (0.56–1.05)	0.00	0.954	0.000

Quality					
High	1	0.80 (0.69–1.01)	NA	NA	NA
Medium	2	1.09 (0.95–1.24)	0.00	0.840	0.000

NA: not available.
